# Supplementary material for: Mechanistic within-host mathematical model of inhalational anthrax
Source: PLoS Comput Biol. 2025 Sep 24;21(9):e1013439. doi: 10.1371/journal.pcbi.1013439 (PMC12459799; doi:10.1371/journal.pcbi.1013439)
Supplement: Attachment — Submitted filename: response to reviewers.pdf [file pcbi.1013439.s001.pdf]

Dear Editor,

Thank you for the time spent handling and considering our manuscript. We would like to thank the three reviewers for the very positive comments on our work, and for having carefully read our manuscript and provided useful feedback. Below, we answer each of their comments and explain how they have been addressed when revising our manuscript. Modifications are highlighted in red in the updated version of the manuscript.

Yours sincerely,
Bevelynn Whaler (on behalf of all authors)

Reviewer 1

"This is an interesting paper that considers an important problem of efficiently modelling within-host dynamics of bacterial anthrax. Using a clear mechanistic structure for disease transmission/progression in different parts of the body, they are then able to not just qualitatively reproduce available data, but to actually fit their results to a number of model organisms, including rabbit, guinea pig, and even a known human outbreak. Overall, the paper is clear and well-written, and I am happy to recommend publication, provided the authors address the following minor issues related to improving clarity/readability."

We thank the reviewer for their positive evaluation of our manuscript.

"1. While units of measurement for different parameters are stated in Table 1, perhaps it would be a good idea to also include them next to parameters themselves in the leftmost column of Table 2."

As suggested, we have included the parameters units in Table 2.

"2. When introducing Sverdlovsk data on p. 13, it is advisable to mention straightaway the limitations of those data in terms of possible use of tetracycline in some patients, but with an unknown start date and unknown duration."

We have now mentioned this limitation in the paragraph in which we introduce the data.

"3. Perhaps, it would be a good idea to mention in Table 3 transitions for PA_{LN} and PA_C in the same way, as they are described for all other model compartments. Also, to be absolutely sure, do I understand correctly that the ODE system representing the mean-field model was not actually used for simulations, and instead it was rather the stochastic version that was simulated using Gillespie algorithm with transition probability as given in Table 3?"

Since the continuous variables PA_{LN} and PA_C do not undergo discrete transitions in the same way as the other variables, we have not included them in Table 3, but have mentioned this in the caption.

We use the Gillespie algorithm to simulate the model at early time points, until the system hits a state where $B_C \geq 1$. Then from this point onwards, we change to using the ODE system for computational efficiency. We have clarified this in the text to avoid confusion.

Reviewer 2

"This is an interesting study on applying a mechanistic mathematical model of anthrax infection to data from experimental infections of animals. The insights from the animal data-based model are also applied toward exploring a model of human incubation time estimates, which is also interesting and useful. The details for the within-host model are novel extensions to prior models in this field, leading to novel conclusions.

While I have not checked all the details of the analysis and results, I have no reason to doubt that they were done correctly. The model and analysis techniques are well explained, the codes to reproduce the analysis are publicly available and appear to be well documented, and the results are plausible and not inconsistent with prior, related work. I think the model and techniques described are quite useful advancements for this area of work."

We thank the reviewer for the positive comments on our work.

"My only suggestion for improvement is regarding the human incubation time data from the 1979 Sverdlovsk infections. The authors use data from a 2006 manuscript by Wilkening, but there was later work that arguably produced a more rigorously derived list of incubation times. A 2012 study by National Academies Institute of Medicine (https://nap.nationalacademies.org/catalog/13218/prepositioning-antibiotics-for-anthrax) cast doubt on some of the incubation times from the Wilkening data set, particularly a few very short (< 4 days) incubation times that were from individuals with unconfirmed Anthrax infection. They judged that a list of incubation times from 31 autopsy-confirmed anthrax victims reported by Abramova et al. (https://doi.org/10.1073/pnas.90.6.2291) provided a stronger evidence base for incubation period distribution from this event.

I wonder if applying the authors' analysis to this set of 31 incubation times would lead to different conclusions compared to the result from the Wilkening data. I suggest the authors attempt this comparison if possible."

We thank the reviewer for bringing this to our attention. We have applied our analysis to the set of incubation times reported by Ambramova *et al.* and have updated this in the manuscript. Encouragingly, this did not significantly change our conclusions. However, we were actually able to better constrain the estimates of the net bacterial growth rate, since large growth rates, corresponding to very short minimum incubation periods, are not consistent with the set of incubation times from Ambramova *et al.*

Reviewer 3

"This was an excellent manuscript to read - very clearly written, solid modelling assumptions, with open and honest interpretation of the work, as well useful suggestions for future work. The authors were able to calibrate their model against experimental data, whilst reflecting on the limitations of their data and approach. Useful insights were generated, but again placed in an honest framework of what more needs to be done before the findings can be used in practice."

We thank the reviewer for the positive comments on our work.

"I have only two very minor points:

- *the assumption that only 1CFU is produced by one chain of bacteria seems odd since the authors describe the chains of bacteria as being very long. Why would only 1CFU emerge from this? There's some discussion of this in the Methods, but a little more discussion here would be interesting."*

  We thank the reviewer for highlighting this interesting point. We assume that 1 CFU emerges from one chain of bacteria because if steps are not taken to break these chains, the bacterial cells in a chain will not have dissociated from each other, and each chain will form a single colony where it lands on the agar media. We have expanded on this in the Discussion.

- *"in Figure 2, two of the data points lie obviously outside of the range of the simulations. Can the authors comment on these? It's not unusual for the simulations to not be able to capture all the data (as is also the case elsewhere in the manuscript), but these two appear more extreme than data points later on that aren't captured."*

  We thank the reviewer for pointing this out. The model predicts that bacteria first reaching the lymph nodes later than 36 hours is a relatively rare event but still a possibility. Therefore, we have increased the number of stochastic realisations shown in Figure 2 from 10 to 20 in order to highlight that the model is able to capture these outlier data points.
